# Supplementary material for: Are carbon emissions trading and green financial instruments synergistic? -Comprehensive quantitative research based on content analysis
Source: PLoS One. 2024 Mar 7;19(3):e0298601. doi: 10.1371/journal.pone.0298601 (PMC10919601; doi:10.1371/journal.pone.0298601)
Supplement: S1 Data — (ZIP) [file pone.0298601.s001.zip › Data/地级市Z_F工作报告.zip!/╕ú╜¿/─■╡┬╩╨/└┤╫╘╣1⁄2╓┌║┼í╛┬φ┐╦╩2╛▌═°í┐_2018.docx]

各位代表：

　　现在，我代表宁德市人民政府向大会报告政府工作，请予审议，并请各位政协委员和列席人员提出意见。

　　一、2017年工作回顾

2017年，在省委、省政府和市委的正确领导下，我市各级政府认真学习贯彻党的十九大精神，以习近平新时代中国特色社会主义思想为指导，围绕“开发三都澳、建设新宁德”中心任务，着力开展“一转三抓比贡献”活动，开拓创新、扎实工作，经济社会发展总体平稳，质量效益明显提升，较好地完成了市四届人大一次会议确定的各项任务。预计全市生产总值1794亿元、增长5.3%；一般公共预算总收入176.85亿元、增长14%，地方一般公共预算收入110.38亿元、增长9.3%；固定资产投资1287.42亿元、增长5.1%；进出口总值305.9亿元、增长20.6%；实际利用外资0.67亿美元、下降71%；社会消费品零售总额565.09亿元、增长10.3%；居民消费价格总水平上涨0.7%；城镇居民人均可支配收入30558元、增长8.5%；农民人均可支配收入14732元、增长9%；税收总收入143.91亿元、增长20.5%，地方级税收收入77.45亿元、增长18.5%，以上两项税性收入和一般公共预算总收入增幅均居全省首位。

一年来的主要工作和成效是：

（一）环三核心区域加快开发。三都澳新区规划建设有序推进。完成总体城市设计及重点地段详细设计国际咨询。建设资金保障取得突破。收储海域面积1.8万亩。后湾、铁基湾两个片区先行启动，金蛇山填海造地加快推进，金后湾、跨海湾围填海项目基本完成。东区污水处理厂加快建设，宁德一中启动新校区规划。北部新城公共服务配套逐步完善。概念性规划完成编制，漳湾片区控规批复实施。锂电新能源特色小镇获批建设。漳湾疏港公路东侨一二标段实现通车，赤鉴湖公园二三期、北区污水处理厂二期和东侨网球中心竣工投用，北山西林学校、福宁路三期、工业路二期等配套设施加快建设。白马片区加速港城联动发展。湾坞溪尾组团控规完成编制。白马港区口岸联检中心、湾坞污水处理厂建成投用。不锈钢新材料特色小镇获批，一期项目开工建设，二三期项目前期工作加快推进。溪南半岛产业区重启建设。园区规划启动调整修编，高科技装备制造园区一期填海造地工程有序推进。宁上高速霞浦至福安段线路规划延伸至东冲半岛下浒镇，溪南傅竹至霞塘段动工建设，沈海高速宁德段扩容工程路线走廊方案进一步深化。

（二）产业结构持续优化。工业发展提质增效。实现规上工业增加值706.73亿元、增长1.8%。出台促进锂电新能源、不锈钢新材料产业发展政策，设立新能源产业基金，两大产业集群实现增加值311.34亿元、增长34%，对规上工业增长贡献率达635.8%。宁德新能源和时代新能源分别成为全球第一的消费类聚合物锂离子电池和锂离子动力电池制造企业，时代新能源入选国家技术创新示范企业，并以其雄厚的研发实力、先进的技术水平和迅猛的发展势头，获得各方面高度认可，社会关注度显著提升。青拓集团完成全国首例跨省钢铁产能置换，400系不锈钢项目启动建设，成为全球最大的不锈钢生产基地，产量占全球10%、全国20%。中铜40万吨铜冶炼项目加快实施，漆包线等深加工项目深入对接。宁德核电5#6#机组具备国家核准条件，霞浦核电示范快堆项目开工建设。广生堂药业获得全国首张替诺福韦乙肝适应症批文，成为唯一拥有两大乙肝一线用药的国内药企。传统产业转型升级扎实推进，取缔“地条钢”企业18家，关停石材企业221家，钢铁行业去产能工作在全省作交流发言。亚南电机、时代新能源与福汽集团联手发展新能源汽车电机电控系统，实现批量化生产；海鸥公司、福鼎城投与省船舶集团合作建造半潜式深海养殖设备；福鼎龙安园区合成革企业全面集中供热，节能减排成效显著。企业微观运行态势总体良好，扣除去产能因素影响，工业用电量增长14.52%，增幅居全省首位。新增高新技术企业11家、国家级星创天地1家和省级重点实验室2家、企业工程技术研究中心1家，发明专利授权227件。现代服务业加快培育。实现增加值617亿元、增长10.8%。全域旅游发展迅速，年接待游客2653万人次、增长20.1%，旅游总收入254.29亿元、增长30.2%。第七届宁德世界地质公园文化旅游节成功举办。九龙漈荣膺国家级风景名胜区，闽东红色旅游系列景区列入全国红色旅游经典景区名录，白水洋、九鲤溪获批国家体育旅游示范基地（项目），寿宁获评全国休闲农业和乡村旅游示范县。消费市场稳定繁荣，新增限上贸易企业141家。电子商务交易额528亿元、增长26%。商品住房销售面积326.48万平方米、增长16.1%，增幅居全省首位。港口物流加快发展，集装箱吞吐量8.58万标箱、增长16.8%，增幅居全省首位。水陆联运中心开工建设，宁德（B型）保税物流中心前期工作加快推进。金融服务业稳健发展，金融机构各项贷款余额1577.53亿元、增长5.6%。出台金融支持实体经济发展5条措施和4个配套政策，省级金融机构加大对我市支持力度，新获得多家金融机构2500亿元授信额度。金融和公检法部门通力协作，金融风险防范和化解工作取得重大突破，金融生态环境持续改善，不良贷款余额从年初98.39亿元降至45.01亿元，不良率从年初6.59%降至2.85%，创2013年以来最低，下降幅度居全省首位，圆满完成了省里提出的“年末不良率控制在3%以内”目标。特色现代农业稳步发展。实现农林牧渔业增加值306.92亿元、增长4.2%。粮食产量64万吨、地方粮食储备15万吨，省市区三级联建的宁德国家粮食储备库迁建项目投用。宁德国家农业科技园区通过验收。食用菌工厂化、标准化栽培模式有效推广，水产、茶叶、果蔬等特色产业加快发展。新增设施农业面积1万亩、全国农村创业创新园区5个和省级家庭农场示范场21家、农民合作社示范社16家，新认定农业产业化龙头企业323家。建成高标准农田17.07万亩。农村土地确权登记颁证进入第三方验收阶段，“两权”抵押贷款余额7.91亿元。

（三）城乡建设统筹推进。中心城区建设管理不断加强。闽东路中段实现双向通车，长期困扰市民的“卡脖子路”问题得到解决。金马北路、金漳路、福利路建成通车，鹤峰路完成“白改黑”。新增公共停车位434个、新能源汽车充电桩240个。署前路棚改一期、金兰小区安置房等棚改项目完成安征迁。市博物馆、档案馆、科技馆和青少年宫“四大馆”开工建设。人民公园等一批园林项目加快改造提升，环东湖慢行道二期建成开放，东湖核心景观区逐步完善。曲尺塘、古溪溪防洪排涝工程建成投用。东湖塘北港完成清淤治理，新建改建一批供水和雨污管网。官昌水库完成主体工程建设。城市管理执法体制改革顺利推进，行政执法区域和权责划分明确。建筑垃圾管理办法制定施行。新型城镇化步伐加快。县域特色经济加快培育，城乡面貌进一步提升。古田国家级、福安省级新型城镇化综合试点工作扎实开展，霞浦三沙光影、屏南药膳、蕉城三都澳大黄鱼小镇深入创建。29个省市小城镇综合改革试点实施项目350个，赛岐、太姥山“小城市”试点经济社会管理和行政执法权限有序下放。福安穆阳、福鼎点头列入全国特色小镇，新入选省级特色小镇创建名单4个。全市完成“两违”综合治理477.77万平方米。县城卫生保洁市场化有序推进，139个“千村整治、百村示范”美丽乡村加快建设；完成939个行政村、3.72万户改厕改水，受益农民41.6万人。改厕工作在全国农村厕所污水治理会议上作典型介绍。综合交通网络加快构建。衢宁铁路宁德段加快建设，宁德北站铁路物流中心、宁南铁路前期工作加快推进。沙埕湾跨海公路通道工程、宁古高速六都复合式枢纽互通动工建设。八尺门作业区3#4#泊位建成投用，漳湾作业区10#、湾坞作业区5#、12#13#等泊位加快建设，城澳作业区6-15#泊位连片开发前期工作有序推进。

（四）改革开放深化拓展。“放管服”改革深入推进，公布“一趟不用跑”和“最多跑一趟”事项819项。调整清理95项市级行政审批和服务事项，市县乡三级权责清单体系基本建立。“双随机一公开”监管机制全面推行。涉企证照登记备案等事项实现“45证合一”。市行政服务中心大楼投入使用，便民利企服务不断提升。国企国资改革深入开展，以管资本为主深化改革转变职能有效推进，取消、下放监管事项18项。财税体制改革不断深化，预决算公开统一平台启动运行，营改增实现行业全覆盖。“1+5”人才政策出台实施，国家“千人计划”人才项目合作交流对接会首次在我市举办。湾坞-漳湾工业园区成为国家首批增量配电业务改革试点，福鼎工业园区、东侨经济技术开发区列入全省首批购售电业务改革试点。国家军民融合创新示范区创建工作扎实推进，国防动员、后备力量建设和海防、人防、反走私工作进一步提升，连续五届获评省级双拥模范城。招商引资成效显著，签约合同项目198个，总投资1226.6亿元、增长5.6%。特别是青拓三元材料、鼎盛精品钢、新能源科技锂电池四期等重大项目有力助推产业延链壮群。实体企业出口实现回升、增长17.4%，其中电机电器、食用菌、锂电池、不锈钢产品及生产设备出口分别增长7.5%、23.3%、62.8%、453.7%。第九届海峡论坛·陈靖姑文化节成功举办，古田临水宫祖庙列入全省首批对台交流基地。闽东北经济协作区宁德联席会成功举办，山海协作对口帮扶持续深化。

（五）生态环境不断改善。环保督察问题整改扎实推进,干部群众环保意识明显增强。水、大气、土壤污染防治深入推进，河长制全面推行，实施水污染防治项目247个，完成安全生态水系建设126.89公里，新建乡镇污水集中处理设施27个、垃圾中转系统48个，主要流域水质优良比例100%。年度能耗总量和强度“双控”目标基本实现，大唐火电超低排放改造通过验收。黄标车淘汰任务超额完成。农用地污染状况详查全面启动，省里下达的工业危废贮存量削减任务顺利完成。领导干部自然资源资产离任审计等4项先行先试改革任务取得初步成果。四个国家重点生态功能区产业准入负面清单完成编制。蕉城水韵九都、霞浦杨家溪入选国家水利风景区，水利风景区创建工作在全国水利风景区建设与管理工作会议上作典型发言。寿宁杨梅洲峡谷晋级国家森林公园、古银硐矿山公园获评国家矿山公园。全市植树造林9.66万亩，治理水土流失23.16万亩，清理海上违规养殖2.9万亩。

（六）民生事业协调发展。精准扶贫成效显著。实现脱贫3.45万人，实施造福工程1.68万人，分别超年度计划7%、18%；完成贫困户危房改造1095户。向1.07万贫困户发放小额贷款5.03亿元，总量居全省首位。为8359名贫困群众报销医疗费用4355.13万元，发放贫困家庭子女助学金和补助6198.35万元、惠及3.25万人次。扶贫工作在全省脱贫攻坚现场推进会上作交流发言。地标精准扶贫在世界地理标志大会上作典型介绍。保障体系逐步完善。全市民生支出237.97亿元、增长17.6%，占公共财政支出80.2%。完成30件为民办实事项目。新增城镇就业3.09万人、农村劳动力转移就业4.06万人，城镇登记失业率2.73%。农村低保标准从每年2650元提高到4000元、惠及7.82万人。2.3万名特定对象享受政府购买居家养老专业化服务。发放城乡居民养老金5.51亿元、高龄津贴4980万元。6250户职工家庭获得公积金贷款27.5亿元。新开工保障性安居工程5198套，基本建成4097套，累计配租配售2.88万套，2025户农村危房完成改造。社会事业补短板扎实推进。新改扩建公办幼儿园、中小学项目43个，新增学位8896个。改善农村义务教育薄弱校358个，新创义务教育管理标准化学校34所。建立宁德民族中学省市县三级共建机制。宁德职业技术学院通过教育部第二轮高职院校人才培养工作评估。宁德师院增设本科专业4个，整合闽东卫校设立二级医学院。分级诊疗制度实现全覆盖，县域内就诊率75.18%、增长14.7%。公立医院院长目标年薪制全面实施，医保管理体制基本理顺。实施医疗机构项目98个，新增床位266张，建成达标一体化村卫生所677个。新改扩建市社会福利中心等养老项目70个，新增养老床位1598张，改造护理型床位2845张，养老服务工程率先超额完成省定任务。蕉城黄鞠灌溉工程入选世界灌溉工程遗产名录。蔡威事迹展陈馆被命名为全国爱国主义教育示范基地。《山哈魂》荣获省百花文艺奖一等奖。新增国家级少数民族特色村寨15个。和谐寺观教堂创建活动深入开展。省运会、老健会筹办备战扎实推进，18个比赛场馆和市老年人活动中心改造提升工程基本完工。我市被推荐为全国文明城市提名城市。社会治理不断深化。“平安宁德”建设持续推进，网格化服务管理中心指挥大厅建成投用。“雪亮工程”建设在全省推进会上作经验交流。反恐维稳和社会治安综合治理措施有力，各类违法犯罪得到严厉打击，群众安全感率达94.62%。党的十九大、厦门会晤等重大活动安保维稳任务圆满完成。福鼎获评全国平安建设先进市。信访态势平稳可控，环境信访工作获环保部通报表扬。安全生产形势持续稳定，食品药品安全保障有力，防灾减灾扎实有效。工、青、妇、计生协、科协、统计、老区、老龄、残联、移民、人事编制、审计、档案、地方志、气象、防震、外事、侨务、对台等工作都取得新进展。

（七）自身建设得到加强。认真贯彻落实市委决策部署，切实履行全面从严治党责任，制定实施党组工作规则和理论学习中心组学习制度，推进“两学一做”学习教育常态化制度化，党风廉政建设和反腐败工作深入开展。自觉接受人大及其常委会监督并报告工作，依法执行人大及其常委会决议、决定，配合开展执法检查、代表视察、专项调研、工作评议和专题询问，办理市人大代表建议348件。支持政协履行政治协商、民主监督、参政议政职能，定期向政协通报情况，重视委员调研协商成果，办理市政协提案342件。主动接受社会和舆论监督。与法院、检察院、工会互动联系持续加强。制定政府规章和规范性文件65件，废止或宣布失效规范性文件91件，依法行政水平进一步提高。机关效能建设持续推进，监察和审计监督不断加强。

各位代表，过去一年是不平凡的一年，尽管经济总量增长受去产能等因素影响较大，但经济运行质量不断提升，产业结构持续优化，为今后发展打下了良好基础。这是中央和省委、省政府正确领导、关心支持的结果，是在市委坚强领导下全市干部群众团结一心、奋力拼搏的结果，是市人大、市政协加强监督、鼎力支持的结果。在此，我代表市人民政府，向全市人民，向人大代表、政协委员、各民主党派、工商联、各人民团体、无党派人士、离退休老同志和社会各界人士，向中央及省属驻宁机构、人民解放军和武警驻宁部队、公安民警，表示衷心的感谢和崇高的敬意！

在肯定成绩的同时，我们也清醒认识到，发展中还存在不少困难和问题，主要表现在：传统产业转型步伐不快，市场竞争力仍然不强；金融生态环境尚需进一步修复，中小微企业贷款难和银行放贷难矛盾仍较突出；区域发展不平衡明显，山海联动有待加强；高层次人才紧缺，人才发展环境还需进一步改善；要素与配套保障还不到位，影响项目建设与投产现象比较普遍；脱贫攻坚存在薄弱环节，精准扶贫、稳定脱贫和规范管理仍需强化；民生社会事业欠账较多，与群众期盼还有不小差距；一些干部思想观念、担当作为、工作效率还不能适应发展需要，等等。对此，我们要高度重视，采取有力措施加以解决。

二、扎实做好2018年工作

2018年是贯彻党的十九大精神的开局之年，是改革开放40周年，是决胜全面建成小康社会、实施“十三五”规划承上启下的关键一年。政府工作的总体要求是：深入学习贯彻党的十九大精神，以习近平新时代中国特色社会主义思想为指导，牢牢把握稳中求进工作总基调和新发展理念，围绕赶超目标要求，坚持以“开发三都澳、建设新宁德”为统领，以提高发展质量和效益为根本，以决胜全面建成小康社会为主线，以产业振兴、项目带动、城乡统筹、脱贫攻坚、深化改革、生态保护、改善民生为重点，全面做好稳增长、促改革、调结构、惠民生、防风险各项工作，保持经济平稳健康发展和社会和谐稳定，续写新时代宁德发展新篇章。

经济社会发展的主要预期目标是：全市生产总值增长8.5%，力争更快更好些；规上工业增加值增长8.5%以上；一般公共预算总收入增长6%，地方一般公共预算收入增长5%；固定资产投资增长10%；出口总值增长5%，实际利用外资增长10%；社会消费品零售总额增长10.5%，居民消费价格总水平涨幅控制在3%以内；城镇居民人均可支配收入增长8.5%，农民人均可支配收入增长9%；城镇登记失业率控制在4%以内；完成年度各项节能减排任务。

实现上述目标，重点要抓好八个方面工作：

（一）突出核心区域，提速三都澳开发

坚持港口、产业、城市、生态“四位一体”联动发展，深入实施“开发三都澳、建设新宁德”行动计划，加快构建环三区域港口群、产业带、城市圈、生态链，形成新的经济增长极。

加快建设三都澳新区。借鉴国内外成熟经验高起点推动新区建设，充分展现滨海风貌和历史文脉，完善生态海湾功能。深化城市设计与控规，编制路网、地下综合管廊等专项规划。用好两支发展基金和政策性银行贷款，推进飞鸾长土岗片区海域收储和铁基湾片区海域市场化配置。动工建设三都澳大道试验段。完成金蛇山填海造地，推动后湾片区学院东路等路网建设，建成东区污水处理厂主体工程。加快宁德一中新校区等标志性项目建设，推出第一批商住项目，示范带动区域开发。

完善提升北部新城。推动区域控规全覆盖，完成七都、八都片区单元控规编制。深入实施新能源特色小镇规划，加快建设福宁北路、工业路、幸福佳园等项目，加强商业和生活服务配套，推动人才、资本等要素集聚，努力打造现代化“产城融合”发展示范区。建成投用北山西林学校一期、七都溪大桥和220千伏岙村、110千伏大厅输变电工程。加强漳湾工业园区基础设施配套，加快漳湾港铁路专用线项目前期，推进漳湾疏港公路蕉城段路面拓宽、东侨三标段建设，建成漳湾10#泊位。

着力打造白马片区。进一步完善海洋功能区划，完成下白石组团控规，优化白马组团产业发展空间。加快实施不锈钢新材料特色小镇规划，推进新城安置房小区、湿地公园和湾坞中小学新校区等15个公共服务配套项目。复工建设白马港铁路支线，推进宁德北至福安湾坞货车联络线前期。加强白马港区港口岸线资源整合、综合开发，发挥青拓集团等龙头企业作用，带动船舶修造、电机电器、港口物流等产业加快发展。

培育发展溪南半岛产业区。理顺开发建设体制机制。加快推进宁上高速霞浦至福安段、沈海高速宁德段扩容工程一期坑门里枢纽互通至霞浦溪南段前期工作，优化扩容工程二期由霞浦溪南至蕉城城澳的路线走廊方案。完成高科技装备制造园区一期等片区吹填交地。规划建设溪南220千伏变电站、杯溪流域供水工程等园区配套基础设施，加快推进岱岐头2个5万吨级泊位前期工作，促进重大项目加快布局建设。

（二）加快转型升级，构建现代产业体系

发展壮大主导产业集群。按照抓龙头、铸链条、建集群要求，加快打造锂电新能源、不锈钢新材料、铜材料以及新能源汽车等产业集群。着力推动锂电消费类电池、动力电池、储能电池协同发展，开工建设新能源科技锂电池四期项目，建成投产新能源三期湖西数码、动力电池项目；加快产业链延伸，开工建设青拓三元材料一期、思客琦、科达利等15个项目，建成投产厦钨正极、杉杉负极、屏南新能源等8个项目。加大风电、光伏发电、调频、微网、船舶等领域锂电产品应用。支持时代新能源申报锂离子电池国家重点实验室。完善不锈钢新材料产业链条，实现200系、300系、400系产品全覆盖。开工建设青拓特钢100万吨铬铁合金、青拓实业850不锈钢冷轧项目，建成投产青拓实业90万吨400系特钢、90万吨高速线材和型材、300万吨工业废渣综合利用项目，形成全市第一个千亿产业集群。推进铜材料产业基地建设，建成投产中铜40万吨铜冶炼项目，延伸拓展漆包线等精深加工项目，争取设立有色金属集散中心。依托锂电新能源产业优势，全力引进知名品牌整车企业和核心配套企业，打造新能源汽车产业链，进一步推进产业结构升级和产业集群间协同发展。

改造提升传统产业。实施50项以上重点技改项目，完成投资45亿元。支持企业加大研发投入，加快培育高新技术企业、科技小巨人领军企业。推动电机电器智能化发展，着力打造“三电”基地，加快推进宁德时代电机新能源汽车电机电控项目建设；加大氢燃料电池系统、关键零配件研发应用，加快布局氢能源市场；支持发展伺服电机、智能发电机组。推动船舶修造向“专精特”转型，发展远洋渔船和深远海养殖装备、海上风电装备等大型钢结构产品，参与军用船舶维修保障服务。开工建设鼎盛精品钢项目，建成投产福华50万吨、荣兴50万吨特钢技改项目。严防“地条钢”违规产能死灰复燃。支持柘荣刀剪产业从普碳钢向不锈钢转型发展，积极承接温州不锈钢加工外迁企业落地。推动合成革绿色改造，完成煤改气工程，延伸发展革基布、箱包等上下游产业。引导化油器发展电喷、通机、汽车配件。建成投产三祥新材1万吨氧化锆项目，推动发展电熔氧化锆系列产品。

积极培育新兴产业。生物与新医药产业要进一步做大做强，落实促进产业发展5条措施，支持安发生物提取技术发展应用，推动广生堂、力捷迅新特药研发，加快太子参产业化发展。旅游业要着眼于全域旅游，加强太姥山、白水洋·鸳鸯溪、白云山等核心景区建设，强化周边特色景点配套，投放共享汽车串点成线，吸引更多游客到宁德休闲游、自驾游、深度游；推动“+旅游”融合，注重旅游产品开发，强化旅游服务标准化，促进向优质旅游转变；办好文化旅游节，加强世界地质公园、霞浦滩涂摄影等品牌建设，加大宣传营销力度，力争全市游客接待量增长20%、旅游总收入增长25%。现代物流业要加快专业化、规模化发展，深化与中远海运、中铁物资等专业物流企业合作。加快水陆联运中心建设，争取宁德（B型）保税物流中心获批。商贸流通业要加强业态创新，引导大型企业开展主辅分离、工贸分离，新增限上贸易企业100家。扶持建设一批区域专业市场。鼓励拓展跨境电商业务，加强京东等大型电商平台“地方馆”建设，扩大农村电商物流配送体系覆盖面。要紧跟新技术革命步伐，积极推进大数据、物联网、人工智能等新兴产业布局发展。

加快发展特色现代农业。落实粮食安全责任制，确保粮播面积196万亩、产量63万吨。探索建立涉农资金统筹整合长效机制，扶持培育一批农业龙头企业，做强水产、食用菌、茶叶、果蔬、药材等特色产业。引导水产养殖向湾外深远海养殖、湾内标准化设施养殖、陆上工厂化循环水养殖转变，建成投产深海智能化养殖设施项目，推动水产品加工规模化。推广食用菌工厂化、标准化栽培和精深加工。加强茶叶标准化体系建设，加大福鼎白茶、坦洋工夫、天山绿茶等品牌推介，推动茶食品、日用品开发。加强农产品、水产品批发市场和物流体系建设，支持富硒富锌等名优特农产品开拓市场。推进三都澳大黄鱼产业园标准厂房、水产品交易市场和三沙台湾水产品集散中心保税仓、冷链物流交易中心建设。加快现代农业示范区、产业园建设，新增设施农业面积8000亩以上，建成农业物联网应用示范点10个。培育农业新型经营主体，新增家庭农场示范场20家、农民合作社示范社30家。实施化肥农药使用零增长减量化行动，加强农资、农产品质量安全追溯监管，新增“三品一标”20个以上。推进防灾减灾救灾机制改革，抓好防汛抗旱、动植物疫病防控、森林防火等工作。

（三）强化项目投资，增强后劲支撑

加强重点领域投资。落实“五个一批”机制，全年计划实施市级重点项目421个，其中在建项目300个。加大组织推进力度，注重晒进度、晒成效，确保年度投资607.2亿元、力争700亿元，开工项目90个、竣工项目70个。加强交通、能源、水利、通信等基础设施投资。推进衢宁铁路宁德段建设。加快宁古高速、沙埕湾跨海公路通道工程建设，建成沈海复线福鼎管阳至柘荣段、福安至蕉城八都段和屏古联络线。推动蕉城城澳至罗源界段、霞浦东冲至火车站段等23个国省干线项目建设。开工建设三都澳深水航道二期、沙埕进港航道工程和漳湾作业区21#泊位，加快城澳作业区1#2#、8#9#、杨岐作业区25#26#泊位建设。推进宁德海上风电项目前期工作，加快霞浦核电、宁德核电5#6#机组和周宁抽水蓄能电站建设。力争开工建设上白石水利枢纽、福鼎管阳溪跨流域引水工程，加快建设赛江防洪三期、闽江古田溪防洪一期等一批水利工程，建成官昌水库。新建续建7个220千伏、20个110千伏输变电工程。完成海岛村全光网建设，全面建成4G网络。

加大招商引资力度。把招商引资作为“一把手”工程来抓，落实县（市、区）主体责任，实施“4+4+X”产业项目招商计划，加强产业链招商、“回归”招商，积极走出去，引进行业龙头企业、发达城市外迁优质企业，确保全年工业项目新招商500亿元。在引进项目的同时更加注重引进先进理念、先进技术和高层次人才。建立市级协调推进机制，盯紧签约、开工、投产三个关键环节，强化安征迁、资金、审批等要素保障，加大闲置厂房、土地盘活处置力度，完善园区基础设施和人才、产业、环境、服务配套，提高签约项目落地率。

激发民间投资活力。滚动策划实施基础设施、公用事业等领域投资工程包，建立PPP项目合理回报机制，吸引民间资本参与。发挥财政性资金带动作用，通过设立基金、政策性融资担保等方式，引导加大实体经济领域投资。加强政银企沟通对接，推进良好信贷市场建设，增强金融服务实体经济能力。打好防范化解重大风险攻坚战，加大金融风险防范化解力度，严厉打击恶意逃废债，加强小额贷款、融资担保公司等机构监管，防控企业债券违约、非法集资和互联网金融等风险，持续优化金融生态环境。规范地方政府举债行为，稳妥处置隐性债务。加强上市后备资源培育，推动时代新能源、盈浩等企业加快上市。

（四）统筹城乡建设，推动融合发展

提升中心城区建设水平。优化总体规划，推进“多规合一”。完成城市开发边界、取土点等专项规划编制，实行控规动态维护。动工建设闽东无线电厂棚改项目，加快建设署前路棚改一期。推进连城路、天德路、南天路、104国道宁古路口至漳湾疏港公路段拓宽等交通干道建设，建成天山东路、南湖滨路等骨干道路，形成畅通南北城区的交通网络。加快“电动宁德”建设，投放一批新能源公交车，建设固定充电桩及移动储能充电设备500个。加快停车设施建设，新增公共停车位400个。建成投用宁德火车站综合客运枢纽一期。新改建一批农贸市场，建成投用肉联厂。动工建设贵岐山污水处理厂技改扩建项目，建成餐厨垃圾处理、建筑垃圾处置中心，完成雨污改造二期，基本消除黑臭水体。建成南港连通渠、南大塘福瑶路至四孔闸段排涝渠。启动大门山公园提升规划建设，加快建设南漈环山栈道、镜台山公园提升等园林绿化景观工程。完成重点区域夜景亮化工程。推进住房制度改革，加强房地产市场研判和价格监管，合理确定土地出让规模与进度，促进持续健康发展。

推进新型城镇化建设。坚持念好“山海经”，统筹县城新区拓展、旧城改造，依托县域资源禀赋和产业基础，加快推进以城促产、强产兴城。加强城乡结合部和特色乡镇建设，完善基础设施服务功能，提升综合承载能力。加快古田国家级和福安省级新型城镇化综合试点建设。推动福安穆阳、福鼎点头两个全国特色小镇建设，实施省级特色小镇创建项目45个、计划投资45亿元以上。加强赛岐、太姥山两个“小城市”试点体制机制创新。推进户籍制度改革，完善重点人群落户政策。

实施乡村振兴战略。打造乡村振兴先行示范市，推进乡村产业振兴，支持发展“田园综合体”，培育休闲农业、定制农业等新业态，促进一二三产融合发展。推进集体产权制度改革试点，发展壮大集体经济，解决427个“空壳村”问题。发挥乡村干部、经济能人作用，带领群众增产增收。推进美丽乡村建设，完成687个行政村改厕改水、惠及35.8万人。推行农村公路“路长制”，加快“四好农村路”建设。推动土地、资本等要素合理配置，促进城乡公共服务均等化、基础设施联通化、产业发展融合化。创新乡村治理体系，推动自治、法治、德治“三治融合”。推进乡村文化传承，弘扬优秀农耕、民俗文化，加强历史文化名镇名村、传统村落和少数民族特色村寨保护发展，守护好乡愁记忆。

加强城市综合治理。加快数字档案馆建设，建成网格化服务管理信息平台和政务云计算中心。深化城市管理执法体制改革，整合住建领域行政处罚权。推进中心城区市场监管执法与城市管理行政执法区域相统一。完成“两违”综合治理370万平方米。推动社区治理共建共治共享，改造提升一批老旧小区，加强历史文化街区保护。加大占道经营、户外广告、高楼违章建筑整治力度。实施城市道路交通文明畅通提升行动计划，加强重点路段路口交通组织，规范共享单车管理，缓解交通混乱、拥堵问题。

（五）注重精准施策，奋力脱贫攻坚

打好精准脱贫攻坚战。加快6个省级扶贫开发工作重点县脱贫进程，确保按照省里的年度脱贫计划序时脱贫“摘帽”。开展第五轮整村推进帮扶，继续实施“六个一”包村、“五个一”包户工程。完成造福搬迁4800人，建成20户以上集中安置点9个。加快农村危旧房屋改造，解决建档立卡贫困户住房安全问题。定期开展“回头看”，及时对返贫、脱贫不稳和遗漏的贫困人口进行再识别，做到应纳尽纳、应扶尽扶，巩固脱贫成果。强化精准扶贫全程监督考核，完善第三方评估机制，规范专项资金使用管理，做到真脱贫、脱真贫。

激发内生发展动力。注重发挥乡村资源优势，因地制宜发展设施农业、林下经济、乡村旅游等特色产业。深化“百企帮百村”精准扶贫行动，推广“企业+基地+贫困户”模式，健全资产性收益扶贫机制，让更多贫困户分享产业发展红利。积极开展订单式培训，增强贫困户创业就业能力。创新金融扶贫产品和服务，力争扶贫小额信贷覆盖面达50%以上。

强化政策兜底保障。加强农村低保制度与扶贫开发政策衔接，提高无业可扶、无力脱贫群众基本生活保障水平。扩大精准扶贫医疗叠加保险政策覆盖面，降低因病致贫返贫风险。实施教育扶贫，提供就学补助、助学贷款，阻断贫困代际传递。引导社会资金设立教育、医疗等各类扶贫基金。完善社会救助、社会福利、慈善事业和优抚安置制度，加大农村留守儿童、妇女、老年人、残疾人关爱服务力度。

（六）深化改革开放，激发发展活力

推进重点领域改革。全面推行网上行政审批服务，加快公共资源交易平台“全市一张网”建设，健全“双随机一公开”监管机制。开展东侨“证照分离”改革试点，深化“多证合一”等商事制度改革，推行全程电子化登记。推进国企国资改革，完善企业内部管控模式，进一步做强做优做大国有资本。加强统计管理体制改革，夯实统计工作基础。开展第四次全国经济普查。完善全口径预算管理制度，加快市县财政事权和支出责任划分改革，强化预算绩效管理。加大涉企税费清理力度，促进实体企业减负增能。落实“1+5”人才政策，实施紧缺急需人才引育工程，坚持本土培养和外来引进并重，让各类优秀人才多起来、用得好、留得住。深化事业单位分类改革。推进管道天然气价格改革，建立居民用气阶梯价格制度。

推动军民融合发展。贯彻落实国家军民融合发展战略，加强向上沟通对接，推进军民融合创新示范区建设。支持优势民营企业申报“军工四证”资格认证，加强军民两用技术项目、产品需求对接，重点推进锂电池、氢能电池、氧化锆等优势产品参与军工配套协作。按照平战结合原则，推进现代化深水大港等基础设施规划建设。加强国防动员、后备力量建设、军地人才培养、双拥共建等工作，抓好军民融合示范点建设。

提升开放合作水平。主动融入“海丝”核心区，开拓“海丝”沿线国家新兴市场，加强海外华人华侨和外国友城交流合作。推进三沙港区口岸建设，推动漳湾作业区水域正式对外开放，加快沙埕湾区域开发。加强口岸“单一窗口”建设，促进贸易便利化。落实外贸出口扶持政策，加强电机电器、水产品、食用菌、茶叶等传统产品出口，扩大锂电池、不锈钢产品及生产设备等新增长点出口。深化与港澳台侨交流合作。办好第九届电博会和第十届海峡论坛·陈靖姑文化节。加快宁台现代农业合作示范项目建设。推进圆瑛、甘国宝、马仙等特色文化和教育、科技、卫生、旅游、体育等对台交流。加强闽东北五市一区、福莆宁和平潭互动协作。

（七）守护青山绿水，筑牢生态屏障

打好污染防治攻坚战。加强环保、海洋督察问题整改。落实河长制、湖长制，推进河岸生态保护、饮用水水源地保护和地下水警戒保护蓝线管理，加强重点流域、湖泊、小流域综合整治。开展海上违规养殖和海漂垃圾专项治理。推进合成革、船舶修造、电镀等重点行业整治，完成县级污水处理厂提标改造。加强挥发性有机污染物控制和火电、冶金等重点企业综合治理，有效应对臭氧污染。加大道路扬尘、建筑垃圾、餐饮油烟等问题处置。全面实施“土十条”，启动工业用地污染状况详查。建成宁德市危废综合处置中心。强化重金属、危化品、核辐射等环境监管。加快创建国家森林城市，加强湿地、石材矿山等生态保护修复，实施造林绿化9.67万亩，治理水土流失18万亩。

创新生态文明机制。落实生态文明试验区改革任务，开展生态文明建设目标评价考核。健全主体功能区制度，严守生态保护红线。落实永久基本农田和城镇开发边界控制，强化海洋自然岸线管控。实行重点流域生态保护补偿，探索生态环境损害赔偿制度。加强企业环境信用评价、控制污染物排放许可制管理。完成环保机构监测监察执法垂直管理改革，健全环保网格化监管体系。

坚持绿色低碳发展。坚决淘汰落后产能，实施节能重点项目10个以上，加快培育循环经济示范试点园区。开展第二次全国污染源普查，执行新建民用建筑绿色标准，落实能源消耗、水资源消耗、建设用地等总量和强度“双控”。开展环境保护费改税工作，加强排污权有偿使用和交易，推进用能权、碳排放权交易改革。推行合同能源管理，加快培育环境治理和生态保护市场主体。

（八）坚持以人为本，增进民生福祉

加大社会保障力度。完善促进就业创业政策，确保全市新增城镇就业2.4万人、城镇失业人员再就业1万人。落实企业工资支付行为守信激励和失信惩戒制度，加快构建和谐劳动关系。推动基本养老、工伤、失业、医疗、生育等社会保险扩面增缴，提高城乡低保和特困救助供养人员护理标准，扩大政府购买居家养老服务覆盖人群。新开工保障性安居工程148套，发放低收入家庭租赁住房补贴476户。

补齐社会事业短板。年度计划投资180亿元，用于民生社会事业领域补短板。新改扩建幼儿园、中小学80所，新增学位2.5万个。创建义务教育管理标准化学校30所。基本完成改善农村义务教育薄弱校任务，缩小城乡、校际办学差距。加强民族中小学建设，深化宁德市民族中学省市县三级共建。推动一批省示范性普高建设。加强基础教育内涵建设，提升教育教学质量。推行教师县管校聘机制，统筹城乡师资配置。推进产学研一体职教园前期工作，加快宁德师院医学院、市教师进修学院研训楼建设，完成宁德职业技术学院二期工程。重视发展特殊教育、老年教育。实施医疗机构新改扩迁建项目151个。建成达标一体化村卫生所700个。加快完善分级诊疗、现代医院管理、全民医保、药品供应保障等制度。做大市医院、闽东医院两大医疗集团，鼓励成立县域紧密型医联体。开展基层医疗卫生服务能力三年提升行动，推动优质医疗资源下沉。加强省市重点专科培育和儿科、产科、精神科等薄弱专科建设。新建续建各类养老项目200个，整改达标乡镇敬老院61个，新增养老床位1000张以上，提高医养结合、居家养老专业化服务水平。同时，继续实施一批为民办实事项目。

推动文化繁荣发展。加快“四大馆”建设，启动宁德大剧院和市工人文化宫、方志馆项目前期，推动基层综合性文化服务中心全覆盖。加强文艺精品创作，深入推进“魅力闽东”文化惠民和文化科技卫生“三下乡”活动。加强优秀传统文化传承发展，推进“七个一批”项目和“十大行动”，贯彻《宁德市畲族文化保护条例》，加快省级畲族文化生态保护区和宁德畲族文化园建设。推进36个红色文化重点项目建设。培育“古村落+文创”、动漫游戏等新业态，打造文化产业“升级版”。

办好省运会老健会。全面完成比赛场馆场地和配套设施建设，精心做好开闭幕式、赛事组织、服务保障、环境整治等各项工作，努力呈现一届富有特色、精彩纷呈的体育盛会。加大优秀教练员、运动员培养力度，强化备战训练，争创比赛佳绩。启动全国文明城市创建工作，深入开展“创文明城、迎省运会”等活动，充分展示闽东人民热情好客、文明有礼的东道主风采。

维护社会和谐稳定。健全矛盾纠纷多元化解机制，提升信访工作法治化、规范化水平。贯彻新修订的《宗教事务条例》，依法加强宗教事务管理。建成天主教闽东教区主教府。加快社会治安防控体系建设，依法打击和惩治黑拐骗、黄赌毒等违法犯罪行为。强化公共安全风险管控，实施公共安全视频监控建设联网应用项目，提高预测预警预防各类风险能力。推进安全生产领域改革发展，严格落实安全生产责任制，坚决遏制重特大安全事故。完成道路安全整治提升。实行烟花爆竹分区域分时段燃放管理。强化食品药品监管，加大“餐桌污染”治理力度。充分发挥工会、共青团、妇联等人民团体作用，做好地方志、气象、防震、档案、老龄、残疾人和关心下一代等工作。

三、切实加强政府自身建设

党的十九大对建设人民满意的服务型政府提出了明确要求。我们要坚持党的领导，加快转变政府职能，发挥市场“无形之手”和政府“有形之手”作用，推进治理体系和治理能力现代化，让行政效率更高、市场主体更活、人民群众更满意。

坚定理想信念。牢固树立“四个意识”，增强“四个自信”，坚决维护习近平总书记在党中央和全党的核心地位，坚决维护党中央权威和集中统一领导。把对党忠诚作为立身之本，严格遵守党章党规党纪，做到信念、政治、责任、能力、作风“五个过硬”，营造风清气正的良好政治生态。推进“两学一做”学习教育常态化制度化，深入开展“不忘初心、牢记使命”主题教育，自觉用习近平新时代中国特色社会主义思想武装头脑、指导实践、推动工作。落实意识形态工作责任制。

坚持依法行政。加强法治政府建设，在党的领导下、在法治轨道上开展工作。完善政府议事规则和决策程序，健全政府法律顾问制度，推进重点领域立法，严格规范公正文明执法，提高运用法治思维、法治方式推动发展的能力。自觉接受党内监督，依法接受人大及其常委会的法律监督和工作监督，主动接受政协民主监督、监察机关监督，认真听取各民主党派、工商联和无党派人士意见，提高人大代表建议和政协提案办理质量。深化政务公开和权力运行网上公开，主动接受舆论监督和群众监督，让干部习惯在提醒和监督中工作生活。

坚决改进作风。传承弘扬“滴水穿石”精神、“弱鸟先飞”意识、“行动至上”作风和“四下基层”制度，真抓实干、马上就办，确保各项决策部署落地见效。加强学习实践，培养专业能力、弘扬专业精神，不断增强推动高质量发展的本领。严格落实中央八项规定实施细则精神和省里《实施办法》，着力查摆形式主义、官僚主义新表现，大力整治表态多调门高、行动少落实差等突出问题，做到纠正“四风”不止步，以钉钉子精神做细做好各项工作。把履职和奖惩结合起来，褒奖敢担当的干部，教育和调整得过且过的干部，提振干事创业精气神。

坚守廉洁底线。认真履行全面从严治党责任，严格落实“一岗双责”，深入推进政府系统党风廉政建设和反腐败工作。构建亲清新型政商关系，培育和弘扬优秀企业家精神，使企业家安心经营、放心投资。完善市场准入负面清单制度，破除歧视性限制和各种隐性障碍，激发市场主体活力。加大审计力度，严密防控项目审批、土地出让、工程建设等重点领域廉政风险，深化标本兼治，保证干部清正、政府清廉、政治清明。

各位代表，进入新时代，面对新使命，我们要全面贯彻落实党的十九大精神，紧密团结在以习近平同志为核心的党中央周围，在省委、省政府和市委的领导下，紧紧围绕“开发三都澳、建设新宁德”中心任务，团结拼搏、攻坚克难，开拓进取、奋发有为，为实现跨越赶超、全面建成小康社会而努力奋斗！
